# Divergent social communication between autistic and non-autistic individuals revisited: unraveled via an Integrated Model of Pragmatic Competence

**DOI:** 10.3389/fpsyg.2023.1248557

**Published:** 2023-11-14

**Authors:** Chang Xu, Tiaoyuan Mao, Shengbin Du

**Affiliations:** ^1^School of Foreign Languages and Literature, Beijing Normal University, Beijing, China; ^2^Institute of Linguistics, Beijing Foreign Studies University, Beijing, China

**Keywords:** autistic and non-autistic language use, divergent social interaction, pragmatic mechanism, Double Empathy Problem, Integrated Model of Pragmatic Competence

## Abstract

Some current studies call for the adoption of the theory of the Double Empathy Problem (DEP) to reappraise autistic individuals' problematic social communications with non-autistic individuals from the perspectives of both sides, rather than exclusively focusing on the social cognition of individuals with autism. However, there is no specific proposal that explicates how such reframed social communications proceed. Herein, we adopt two subcomponents of the Integrated Model of Pragmatic Competence (IMPC) to clarify the main factors leading to the divergent social interactions between the two groups. Internal Pragmatic Competence (IPC), revealing how they both independently think about internal linguistic and communicative issues, echoes DEP's reference to different mindsets and elucidates why uncooperative social communications happen. Pragmatic Competence for External Communication (PCEC) explains how the impaired communications among organism-internal submodules and/or their unsuccessful interactions with outside contexts impede the external sociopragmatic communications between the two sides. Put together, the operation of the two components helps to interpret the cognitive pragmatic mechanism underlying social communications and suggests a potential holistic perspective to improve such communications in terms of both sides.

## 1. Introduction

Generally, people with autism spectrum disorder (ASD) are burdened with problematic social communication and interaction, such as persistent sociopragmatic inadequacy, and labeled with repetitive and stereotyped behaviors, interests, and activities, which limit or impair everyday functioning (American Psychiatric Association, 2013). In the past few decades, cognitive psychologists, clinicians, linguists, and neuroscientists, among others, have conducted comprehensive cross-disciplinary research on ASD (cf. Kissine, 2021; Lord et al., 2021; Mao, 2023, a.o.). This type of interdisciplinary investigation, with the purpose of delving into the nature of autism in depth, makes use of powerful contemporary scientific methods and advanced technologies to reveal the characteristics of various aspects of ASD, including social, cognitive, developmental, and genetic dimensions. Based on what has been done, it seems proper to say that the research on ASD mainly focuses on “a particular psychological and (neuro)biological state with its own known features” (Kana, 2022, p. xiii).

Along this vein, within the cross-disciplinary research of ASD, one of the common perspectives taken when investigating the cognitive or behavioral profiles of autistic people is the cognitive deficit view of autism.^1^ In general, the cognitive investigation of normal social communications focuses on the cognitive processing of various types of information in corresponding sociocultural contexts, such as linguistic cues and encyclopedic knowledge. In López's (2022, p. 368) words, this type of investigation means expounding “social cognition,” which is understood as “a set of neurocognitive or psychological processes underpinning individuals' abilities to process, interpret, and respond to social signals to make sense of others' behavior during social interaction” (Frith and Frith, 2007, p. 724–728; Arioli et al., 2018, p. 1). Thus, due to the opaqueness of the human brain in terms of information processing, the investigation on social cognition in typical situations maintains that communicators must read each other's minds so as to reason out the semantic propositions and pragmalinguistic-sociopragmatic meanings, otherwise a failed or infelicitous sociocultural communicative interaction will occur. Accordingly, given the turbulent interactive communication performed by autistic individuals, the deficit view of autism assigns the problematic social interactions to a deficit in autistic individuals' “Theory of Mind” (ToM) ability, i.e., “the cognitive ability to attribute mental states to other people” (Goldman, 2012, p. 402)^2^; furthermore, the dysfunction of the ability is accredited to corresponding neurobiological disorders [see Kana (2022) for a review]. In the existing literature, even if this one-sided view sounds a little extreme, it does not entirely lack empirical or clinical support since some researchers have found that autistic people have difficulties reading the inner minds of non-autistic people (Kissine, 2012; Sheppard et al., 2016; Andrés-Roqueta and Katsos, 2017).

Recently, Kissine (2021), proceeding from the sociopragmatic profile of highly verbal autistic individuals, took the impaired intrinsic link between mind-reading and language use as evidence to unravel the controversies between Constructionism and Nativism in terms of language acquisition and its use.^3^ Specifically, the evidence from experimental studies on pragmatics in autism denotes the separation of many autistic pragmatic processes from reading conversation partners' perspectives and the divorce of language learning or the emergence of languages from intersubjective communication. Therefore, the linguistic profiles of autistic people seemingly support Nativism's assumption of an internal mechanism for language use and acquisition but not Constructionism's advocation of solely intersubjective language use and merely usage-based language acquisition. In this vein, Mao (2023), under the framework of IMPC (cf. Mao, 2020, 2021; see Section 3), demonstrated why mind-reading could not necessarily be considered the only mental support for “egocentric” or non-interactive autistic individuals when they conduct abstract thinking activities, but its dysfunction or absence definitely renders authentic sociocultural communication impaired. In brief, one component of IMPC, i.e., the internal part of pragmatic competence (IPC), accounts for the reason autistic people do not rely on mind-reading but on self-sufficient mental interactions among organism-internal submodules (i.e., syntax, semantics, pragmatics, and phonology-phonetics) within the language module or on nearly intact grammatical submodules to process linguistic and communicative needs at the abstract-thinking level (for more detailed explanation of IPC see Section 3). The other component of IMPC, viz., the pragmatic competence for external communication (PCEC) that is indispensable for facilitating the external authentic intersubjective language use, elucidates why the dysfunction of modular interactions within the language module and their unsuccessful interactions with outside contexts result in autistic individuals' sociopragmatic impairment (see Section 3 for details of PCEC).

However, Milton (2012, p. 886) called for a reconceptualization of autism as a condition that is “both biologically and socially derived”, and he especially ascribed the divergent social interactions between autistic and non-autistic people to DEP first proposed by him. That is to say, it is “the mutual incomprehension that occurs between people of different dispositional outlooks and personal conceptual understandings when attempts are made to communicate meaning” (Milton, 2013) that brings about the unsuccessful communicative interactions between autistic and non-autistic individuals, rather than only blaming the cognitive inadequacy on the side of autistic people. The understanding of DEP has since been further examined. For example, Mitchell et al. (2021) stressed the necessity to explore the relationship between mental health and the perceptions of the neurotypical majority under DEP since the misperceptions of the neurotypical majority influence the perception and behaviors of autistic people. Moreover, López (2022) pointed out that the cognitive ability to process social information is only one element contributing to the understanding of others, and it is much better to adopt “the second-person approach” (Di Paolo and De Jaegher, 2016) and center on the social interactions themselves that are key to understanding others. That is to say, the presence of social partners influences communicative behaviors, and social interactions should be reevaluated from the perspective of both parties of social communications, as suggested by DEP. In this vein, DEP seems to be primarily engaged in the interaction between high-functioning individuals with autism and non-autistic people (since one reviewer points out that certain severe forms of intellectual disability in autistic individuals might render interactive communicative activities difficult to sustain).

Even though the current research makes use of the principles of DEP to investigate the divergent sociopragmatic communications between autistic and non-autistic people from both sides, the nature of the divergence of such unsuccessful social interactions is still unclear (see next section). Given the shared target of DEP and IMPC, viz., addressing why autistic people have difficulty tuning into cooperative communication with non-autistic partners and vice versa, it is significant to elaborate on which cognitive pragmatic mechanism(s) within them can influence the communications between autistic people and their non-autistic counterparts. Furthermore, given DEP's object of attention, it seems more practical for IMPC to be primarily limited to investigating the interactive communications between high-functioning individuals with autism and non-autistic people (even if it can offer explanations for other situations). To serve this purpose, we first sketch the cause of DEP and its action plan, and then explain how to deconstruct typical or atypical communicative interactions via IMPC, which paves the way to finally decode DEP's concern on the divergent social interactions between autistic and non-autistic people.

## 2. The cause of DEP and its solution

Basically, as aforementioned, research on social cognition has attached prime importance to the ToM approach (i.e., understanding others by discerning their mental states). However, this approach neither values the role of social interactions when processing social information nor places communicators in the *actual* social interactions (López, 2022). Against the philosophy of ToM, DEP insists that unsuccessful sociopragmatic interactions between autistic and non-autistic people cannot be attributed to autistic cognition alone but to the double empathy gap between the two sides (Milton, 2012). In other words, since autistic and non-autistic people have different experiences of the world, they struggle to empathize with each other in social interactions. In this case, the empathy problem takes the form of “a two-way street” (Hacking, 2009), giving rise to a bi-directional discrepancy in communicative styles and a failure in reciprocal understanding (Milton, 2013).

Given that the double empathy gap engenders a breakdown in mutual understanding and therefore in social interactions, Milton (2013) took “shared interactional expertise” as the indispensable requirement to fill the gap. Following the explanation of the nature of communicative expertise (Collins and Evans, 2007), the shared interactional expertise, either as a competence, social practice, or something inherent in persons, should be acquired so as to bridge the double empathy gap that is detrimental to reciprocal understanding (Milton, 2013). Ideally, in Milton's proposal, if autistic individuals can gain enough interactional expertise, they could potentially “pass” as non-autistic. Simply put, social interactions could become smooth if both autistic and non-autistic people share in the same sociality (even with somewhat different communicative ways).

However, even though the shared interactional expertise seems to be a means to resolve the double empathy gap and redefine the role of autistic and non-autistic people in social communications, it is unclear which specific subcomponent(s) the interactional expertise consists of and how it facilitates the reciprocal sociopragmatic interactions from both sides of autistic and non-autistic people. This being the case, a precise proposal is needed to decode the basic properties of such expertise that underpin the social interactions between autistic and non-autistic people; one that also seeks to understand how the construct of that expertise would explicate language use between the two sides.

## 3. Untangling (non)divergent communicative language use via IMPC

The importance of a two-sided approach to understanding the divergence that exists within sociopragmatic interactions of autistic and non-autistic people has been intensified by a recent critical review of autism and other neurodevelopmental disorders' research spanning the past 60 years. Specifically, the *Lancet* Commission on research in autism points out that valuing both autistic individuals' own preferences or needs and other neurodevelopmental conditions concerned will benefit society as a whole (Lord et al., 2021). Without a doubt, this proposition aligns with Milton's basic tenet for advocating DEP, i.e., exploring autism from the “biological and social perspectives” (Milton, 2012, p. 886). In this case, IMPC, a theory-neutral proposal of pragmatic competence, appears to be capable of explaining why the double empathy gap impedes language use and/or comprehension between autistic and non-autistic populations because IMPC is deeply rooted in the biological endowment of the Faculty of Language in Chomsky's (2005) sense and is compatible with theories of social communication and cognitive mental processing, including ToM (Mao, 2023).

**Figure 1:**
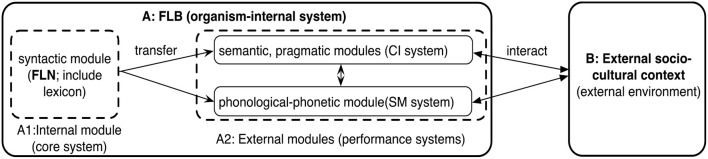
Interactions in FLB and sociocultural contexts [FLB, the Faculty of Language in the broad sense; FLN, the Faculty of Language in the narrow sense (see Hauser et al., 2002)].

Essentially, IMPC bases itself on the complementary relationship between grammatical and pragmatic competence, which can be traced back to Chomsky's (1977, p. 3) dichotomy of grammatical and pragmatic competence as “two components of the attained cognitive state”.^4^ Under this design of language architecture, the interactions presupposed by IMPC among organism-internal cognitive submodules and their interactions with outside contexts come very naturally, as Figure 1 shows (Mao, 2020, 2021).

In detail, the specific interactions, as indicated in Figure 1, have been endowed with neurobiological and evolutionary evidence. For instance, the cerebral anatomical or functional submodules or innate neural structures cooperate tightly with each other in linguistic computations (Pléh, 2000; Friederici, 2017). Moreover, the brain rewiring that originates from certain slight neurological mutations triggers the formation of the binary combinatorial operation Merge in a short time window of human evolution (Berwick and Chomsky, 2016; Chomsky, 2023). Thus, the significant change has built up the engine of generative procedure in the human language system, outputting structural representations as information or computational instructions for the Conceptual-Intentional (CI; semantic-pragmatic) system to carry out thought, interpretation, and organization of action, and for the Sensory-Motor (SM; phonological-phonetic) system to externalize for production or assign to sensory data for perception (Chomsky, 2015; Mao, 2023). In this case, a modular view of language and language use is neatly exhibited in Figure 1.

Thus, taking the cross-modular interactions as a departure, the dynamic interactions in Figure 1 present an explicit route map for linguistic computations, unveiling how language is used to realize both abstract human thoughts and authentic communications. In other words, these two types of language use in the daily life of autistic and non-autistic people, viz., for pure thinking activities and authentic sociocultural communications, clearly reflect the two-side-of-one-coin attributes of human language (i.e., thought and communication) (Mao, 2020). In this case, the boundary of IMPC is strictly defined. To reveal both the dual properties of language use and the attributes of human language, two corresponding subcomponents should be established within IMPC.

In consideration of the first subcomponent, the interactions among organism-internal linguistic or cerebral submodules for thought in the mind/brain demand the activation of an internal part of IMPC, i.e., the Internal Pragmatic Competence (IPC). That is to say, IPC employs the interactions among organism-internal linguistic submodules and/or their interaction with outside worlds to execute pure linguistic computations and think about internal “silent” sociocultural communications at the abstract thinking level (i.e., realizing a kind of abstract language use for thought), such as syntax-semantics interaction (A1 → A2 in Figure 1) and the interactions between syntax-semantics-pragmatics computations and outside contexts [(A1 → A2) ↔B in Figure 1], respectively.^5^ To illustrate the operation of IPC, we can take the interaction between syntactic and pragmatic submodules as an example. For instance, in a tea party, an old Japanese male guest talked to another old Japanese female guest, as (1) shows.

(1)  また     病
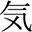
に            なった   の?
 again    sick-dative    became    SFP(Question)
“Did      you feel         sick         again?”

When the old Japanese female guest heard the question sentence (1), how did she reason out the implied pragmatic meaning? In general, she must be familiar with the syntactic structure and reach the literal meaning of the male guest's utterance because the syntactic representation and literal meaning function as a foundation for the unfolding of IPC. Therefore, she first rebuilds the syntactic structure based on the lexical items that she obtains by decomposing the sound flow of the male guest and then she forms a plain propositional meaning on the basis of the syntactic representation generated by the syntactic submodule, that is, “彼女がまた病
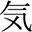
になったかどうか(知っていますか”(whether she feels sick again), as described in a logical form below.

(2)     λP. P*ϵ*                    {a.彼女はまた病 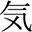
になった;    
    (Intended: λP. Pϵ    {a. she felt sick again;
  b.彼女はまた病
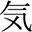
になったのではない}.
  b. she did not feel sick again})^6^

Starting from the propositional meaning (2), she relies on IPC to establish the relevancy between particular linguistic symbols and their corresponding pragmalinguistic knowledge to obtain the indirect meaning. Specifically, by IPC, she activates the relevant pragmatic knowledge from encyclopedia knowledge or pragmatic submodule for “また” (again), that is, “to suffer from something one more time”. Based on it, she further reasons out the possible intended meaning of the male guest—“she is in poor health condition”, as formally described in (3).

(3)     λ x.                       (xが体
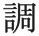
不良です)    
    [Intended: λ x.       {x is in poor health condition}    
    (g). (“g” denotes    “彼女”)    
    (g). (“g” denotes    “she”)]^7^

In this vein, through the successive computations across modular interactions presupposed by IPC, namely, from syntax through semantics to pragmatics (cf. A1 → A2 in Figure 1), the female guest can abstractly think about or reason out the pragmatic meaning embedded in the syntactic structure.^8^

As such, the cross-modular interactions featuring “invisible” IPC display how human language is used to reason out indirect pragmatic meanings at the abstract-thinking level (for more exemplifications see Mao, 2020, 2021, 2023). In this sense, the assumption of IPC is capable of clarifying the linguistic profiles of autistic individuals since they can independently process indirect meanings and acquire languages without solely relying on intersubjective language use (for the same view see Geurts et al., 2020; Kissine, 2021; Mao, 2023). Naturally, such independent thinking is also available for non-autistic people. Therefore, under the proposal of IPC, it seems possible to explain why the incongruent “two-way-street” social interaction occurs between the two sides.

As for the second subcomponent, the other part of IMPC, viz., Pragmatic Competence for External Communication (PCEC), also requires utilizing the cross-modular interactions among organism-internal linguistic submodules and their interactions with outside worlds to realize external authentic sociocultural interactions (i.e., conducting a kind of authentic language use for sociocultural communication). For example, the normal unfolding of PCEC resorts to the interactions between syntax-semantics-pragmatics and outside authentic contexts and between syntax-phonology-phonetics and outside authentic contexts, i.e., (A1 → A2) ↔B in Figure 1 (for more demonstrations see Mao, 2020, 2021, 2023). Along this vein, PCEC neatly underlies the use of externalized linguistic representations from organism-internal linguistic submodules in authentic sociocultural occasions. In other words, PCEC, the “visible part” of the innate disposition for communicative interactions in the sense of Grice (1975) and Kissine (2021), facilitates interlocutors to adapt to partners' declared perspectives or read their minds, and then obtain useful clues to reason out literal and non-literal/indirect meanings. In this situation, given that getting involved in authentic sociocultural communications in a fully interactive or intersubjective manner is a weak point of autistic individuals (see American Psychiatric Association, 2013), PCEC, referring to the established operative route map shown in Figure 1, seems to be able to offer an explanation for why autistic individuals encounter unsatisfactory or unsuccessful sociocultural communications. Put another way, either the dysfunction of interactions within organism-internal submodules (i.e., A1↮A2) and/or their failed interactions with outside contexts [i.e., (A1 → A2)B or (A1↮A2) ↮B in Figure 1] will result in the incongruent social interactions. By the same token, a lack of pragmatic information can also make non-autistic individuals' communication with their autistic partners unsuccessful.^9^

**Figure 2:**
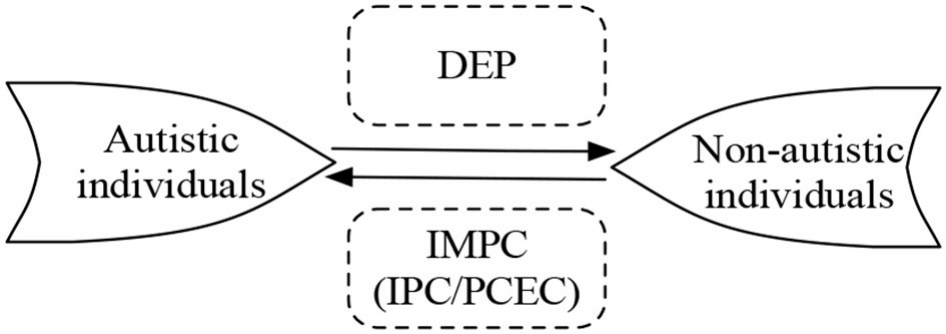
Theoretical reciprocity between DEP and IMPC during the explanation of interactions between autistic and non-autistic individuals.

To sum up, it might be safe to say that, for both autistic and non-autistic people, abstract thinking activities and authentic sociopragmatic interactions fall precisely within the explanatory domain of IMPC. Indeed, within IMPC, IPC and PCEC can clarify why autistic and non-autistic language users are non-intersubjective and interactive/intersubjective, respectively, in corresponding occasions (cf. Mao, 2023). Armed with this idea, we can make a prediction about the relationship between DEP and IMPC when investigating the unsuccessful communicative interactions between autistic and non-autistic people. In other words, based on IMPC, the reason there are unsmooth communicative interactions between autistic and non-autistic partners, aligning with the primary concern of DEP, could be delineated by IPC and PCEC, respectively. Along this line, it might be intriguing to apply this proposal from within IMPC to decode what DEP implies for the inadequate social interactions in terms of both autistic and non-autistic people, rather than solely from the role of autistic individuals, as shown in Figure 2.

## 4. Decoding DEP's concern on divergent social communications between the two sides

As is known in the literature, no matter whether researchers favor applying ToM or DEP to explore the characteristics of autistic social communications, they both recognize that autistic individuals have deficient social interactions with non-autistic counterparts. However, the main difference between the two avenues of research lies in the fact that those subscribing to ToM blame the unsuccessful sociocultural language use exclusively on autistic individuals' failure to align their mental states to their partners, while those believing in DEP criticize some researchers for the biased mindset on autistic individuals' social cognition, such as attributing autistic individuals' unsuccessful understanding of the mental states and motives of other people to the neurological disorders or failure in application of empathy to interlocutors as “neuro-typical” individuals do in normative psychological models of human interaction (Milton, 2012, p. 883–884). As a result, they stress the reinvestigation of autistic individuals' behavioral traits and the correction of the stereotype of autistic individuals being unexpressive during their interaction with non-autistic counterparts (cf. Milton, 2012; Mitchell et al., 2021).

Given the contradictory views on the social communication between autistic and non-autistic people, IPC stands out naturally as an eligible means to decode why the double empathy gap results in divergent communication between autistic and non-autistic individuals. As alluded to earlier, the basic idea of DEP is characterized by mutual incomprehension and collapsed reciprocity that blocks understanding due to the different life experiences and dispositional outlooks of the two groups. In this vein, the divergent personal needs and expectations, together with communicative manners (e.g., egocentric vs. cooperative), hinder the necessary information from being successfully communicated from autistic to non-autistic people and vice versa. This situation directly conforms to IPC's delineation of the independent status of thinking agents within internal abstract-thinking activities. Hence, as suggested by IPC, it is reasonable for autistic individuals not to refer to others' mental states and instead to conduct their own silent thinking for internal pure linguistic computations and sociocultural communicative issues in real or imagined contexts. That is, they can realize a kind of abstract language use. Meanwhile, from the perspective of non-autistic communicators, they are also able to make use of their own IPC to abstractly think about both linguistic and communicative issues. In this way, both autistic and non-autistic people maintain their own independent communicative manners in the closed loop of their inner minds, which thereby induces incongruent silent communication. To illustrate this tendency, let us examine one example involving understanding non-literal hyperbolical meaning. For instance, a caretaker (Part A) talked with a child with ASD (Part B) at a family gathering (adapted from Geurts et al., 2020).

(4)   Part A: *Does this schoolbag weigh a ton?*
    Part B: … (no reply)

In (4), the conversation is incomplete or unsuccessful since Part A (non-autistic) does not have an explicit reply from Part B with ASD. In this instance, does it mean that Part B did nothing in the conversational turn? The answer seems no. Basically, there are two possibilities for this situation. On the one hand, due to the different expectations or needs, Part B primarily ignores what Part A says and just thinks about other matters that attract her/him, without responding to Part A (no mind-reading). Therefore, in dialogue (4), the interactions between syntax-semantics-pragmatics computations and outside contexts collapse, such as (A1 → A2) ↮B in Figure 1.

On the other hand, as indicated in Geurts et al. (2020, p. 124), autistic children with low verbal ability can comply with indirect requests, such as “You forgot the water in your bag.” (Intended: “Go and fetch the water from your bag.”). Accordingly, we can conclude that Part B can abstractly think about the semantic proposition and pragmalinguistic meaning on the basis of the syntactic structure that Part A generates via Merge but completely disregards Part A's intention without uttering a word.^10^ This is totally against what is required in cooperative communication. Thus, the interactions between syntax-semantics-pragmatics computations and outside context experience the same failure as the first possibility, namely, (A1 → A2) ↮B in Figure 1. In detail, following the route map of cross-modular interactions underlying the operation of IPC, Part B first sets the foundation for IPC to unfold. That is, utilizing syntactic and semantic competence to deconstruct the syntactic structure built by Part A and then form its literal proposition: “whether the schoolbag weighs a ton.” Further, Part B selects from her/his background knowledge the corresponding relevant pragmatic knowledge that is relevant to certain syntactic constituents and puts it into the pragmatic submodule to reason out the non-literal hyperbolical meaning.

Crucially, to reason out the hyperbolical meaning “the schoolbag is very heavy” via IPC by means of “the abductive reasoning” (cf. Mao, 2022), Part B must exceed the literal meaning of “weigh a ton” and activate or match the relevant pragmatic knowledge of “weigh a ton”, that is, “as heavy as an object of 1000 kilograms.” In this case, facilitated by IPC, Part B makes use of the relevancy between the syntactic constituent “weigh a ton” and its corresponding pragmatic knowledge, and reasons out the hyperbolical meaning—“very heavy”. In this process, Part B conducts the cross-modular pure mental processing to reach the pragmatic meaning without reference to outside sociocultural contextual cues, along with the egocentric communicative manner of autistic individuals that blocks their explicit responses. Also, as for Part A, she/he proceeds with the “silent” abstract mental processing for the reason Part B offers no reply or for other matters. As such, because of both sides' adhering to their own independent internal thinking, even if it is possible for both sides with different perceptions of the world to struggle to understand or empathize with each other, the double empathy gap is the inevitable result.

Interestingly, the social interactions of this kind fairly match Chomsky's (2011) reflection of the property of abstract thinking activities on various occasions. In Chomsky's discussion, the thinking activity can be regarded as a kind of “silent communication” even though the term “communication” treated in this fashion could be deprived of significant conversational partners. The reason for this type of reinterpretation of “communication” is that language use is “overwhelmingly internal from a statistical perspective, i.e., speaking to oneself” (Chomsky, 2011, p. 266), either in authentic or imagined contexts. All in all, if IPC's elucidation of the deficient social interactions between the two sides is on the right track, it is no wonder that the empathy gap that originates from different conceptualizations of the world and behavioral manners brings about the unsmooth social communications between autistic and non-autistic individuals.^11^

In addition, the crucial concern of DEP, i.e., reevaluating autistic individuals' ineffective interactions with non-autistic partners in authentic communications, can be cleared up within the explanatory force of PCEC. In general, under DEP, it is the sociocultural communicators possessing different personal dispositions or the “differently disposed social actors” (Milton, 2012, p. 886) that give rise to the social interaction breakdown. That is to say, the absence of efficient bi-directional relevant information renders both autistic and non-autistic people unable to enter cooperative interactive tracks. Be that as it may, with the establishment of IMPC, the cause of the failure becomes clearer under its postulation of PCEC. In detail, the deficiency of relevant information, either linguistic or paralinguistic, is unable to propel the social interactions between the two groups, which unsurprisingly leads to unsuccessful reciprocal communications. As a result, for both sides, the lack of relevant contextual information drastically inhibits the fluent interactions between organism-internal submodules and outside sociocultural contexts, viz., (A1 → A2) ↮B in Figure 1. Take one authentic dialogue between the two groups from *The Curious Incident* [a novel about autism. cf. Semino (2014)] to exemplify the circumstance. Specifically, in that scene of the dialogue, Christopher is questioned by a police officer who wants to know which person killed Mrs. Shears' dog (Mrs. Shears, Christopher's neighbor, called the police after discovering Christopher who was holding the body of her dog with a garden fork sticking out of its stomach in her garden in the middle of the night).

(5)    The policewoman: *Would you like to tell me what's       going on here, young man?*
    Christopher (with ASD): *The dog is dead*.

In this authentic scenario, Christopher is expected to answer the policewoman cooperatively when faced with the questioning. In fact, the reply of Christopher is insufficiently informative or irrelevant since the propositional meaning of Christopher's utterance—λ x. (x is dead) (d). (“d” denotes “dog”)—is not congruent with what the policewoman wanted to know, viz., why the dog was dead. Basically, the infringement of Gricean conversational maxims is regarded as expressing speakers' intentions, such as deceiving and triggering implicatures. Yet, Christopher's breach of the Quantity sub-maxims (as informative as is required) seems to convey no such intention but lies in his inability to assess what the partner needs to know (Semino, 2014). Under the assumption of PCEC, it is possible to offer a more specific explanation of why Christopher makes such a divergent reply. To wit, even if the cross-modular interactions within the organism-internal submodules are intact in driving the linguistic computations underpinning by any abstract thinking, such as (A1 → A2) in Figure 1, the sociopragmatic insufficiency that is persistent among autistic individuals like Christopher prevents him from adapting to the outside sociocultural contextual needs, as revealed by (A1 → A2) ↮B in Figure 1. In this circumstance, although both autistic and non-autistic individuals strive to empathize with each other as DEP requires (even in a passive way since they are forced by certain urgent communicative goals), the gaps, caused by the inconsistent dispositional outlooks or the like, block the exchange of necessary relevant messages that are bound to manifest in the context for facilitating smooth interactive communications. Accordingly, there will be no easy social interactions for both sides.

Moreover, the aforementioned scenario will even become much worse in certain extreme circumstances. For instance, with certain specific language impairments occurring in more than one or all the linguistic submodules shown in Figure 1, such as syntactic and semantic deviations in the syntactic and semantic submodules respectively, the dysfunctional interactions among distinct organism-internal submodules and their unsuccessful interactions with outside sociocultural contexts, viz., (A1↮A2) ↮B (cf. Figure 1), will yield completely crashed sociopragmatic interactions between autistic and non-autistic people. In this case, if ASD is gradually alleviated to some degree by means of neurobiological rehabilitation and medical or holistic treatment (McIntyre et al., 2020), social interactions can be recovered step by step. Meanwhile, it is also significant for non-autistic individuals to create a friendly conversational environment with positive linguistic or paralinguistic means and show more patience and empathy for their conversational partners with autism. For example, non-autistic communicators can use more genial paralanguages, like facial expressions and gestures, to make the information or intention well communicated between the two sides. These practices, on the one hand, will help autistic individuals dismiss the feeling of being socially excluded and ignored in interactive communications; on the other hand, the social needs of autistic individuals are carefully addressed in a harmonic way. In this way, the double empathy gap can be mitigated somewhat because both autistic and non-autistic individuals will break through the hurdle that causes insufficient sociopragmatic communications and can adapt to each other's perspectives and/or intentions. In this situation, apart from the above explanation of the mechanism of the social interaction itself, the independent characters of interlocutors can be preserved at the same time according to PCEC (or more broadly, IMPC), thus satisfying both the cognitive and social requirements of DEP.

## 5. Conclusion

The present study addresses the extent to which the concept of DEP on autistic and non-autistic language use can be explicitly reinterpreted via IMPC, a newly constructed pragmatic mechanism that underlies both autistic and non-autistic cognitive pragmatic processing for both pragmalinguistic and sociopragmatic language use. Within IMPC, IPC facilitates autistic and non-autistic populations to abstractly think about the internal linguistic and sociocultural communicative issues in their own independent ways (a kind of abstract language use for thought), revealing the reason mutual incomprehension leads to the double empathy gap and then to divergent social interactions. PCEC makes it clear that the dysfunction of cross-modular interactions and/or their unsuccessful interactions with outside contexts triggers the double empathy gap, eliciting mismatched or collapsed sociopragmatic interactions (a kind of authentic language use for communication). These analyses tentatively demonstrate a pathway for how to realize DEP's expectation of bridging the incongruent “two-way-street” language use between autistic and non-autistic people and treating autism as a condition that is both biologically and socially derived. In addition, future analyses that follow IMPC might provide a basis for designing a language therapy method and figuring out holistic means in terms of autistic and non-autistic people to improve sociopragmatic communications between the two sides, which warrants further exploration.

## Data availability statement

The original contributions presented in the study are included in the article/supplementary material, further inquiries can be directed to the corresponding author.

## Author contributions

TM designed the research. CX and TM wrote the paper. CX and SD discussed and revised the paper. All authors contributed to the article and approved the submitted version.

## Footnotes

^1^As one of the reviewers points out, the Theory of Mind or social cognitive hypothesis of ASD is only one of a few competing hypotheses, and other models related to autism research, such as “deficits of executive function” and “weak central coherence”, should be mentioned. We agree with the reviewer and summarize the key points here. Basically, executive function facilitates people to make plans or decisions and control emotions or impulses based on their experience of the world. In Hughes et al.'s (1994, p. 477) words, the “executive function is an umbrella term for the mental operations which enable an individual to disengage from the immediate context in order to guide behavior by reference to mental models or future goals.” In this case, the executive dysfunction in autism affects autistic individuals' communication, verbal reasoning, and daily tasks, etc. As for “weak central coherence”, Frith (1989, p. 97), based on her concept of “central coherence (pulls together large amounts of information)”, argues that “without this type of high-level cohesion, pieces of information would just remain pieces, be they small pieces or large pieces.” Therefore, owing to the “weak central coherence”, people with ASD normally attend to and/or remember details instead of global forms or meaning, which might subsequently bring about divergent communication.

^2^As Sabbagh and Bowman (2018) noted, ToM (Theory of mind) is often called a “theory” because although one cannot see others' mental states, one can hypothesize their existence and make probabilistic judgments about their specific contents based on a range of relevant evidence. That is to say, it is a kind of cognitive ability to discern the mental states of other people (Mao, 2023).

^3^Constructionism mostly “relies on classic Gricean reconstructions of human communication as a coordination problem, to which building a model of the speaker's mental states may provide an optimal solution” (Kissine, 2021, p. 141). In this case, constructionists purely insist on the intersubjective view on language use and acquisition (cf. Tomasello, 2000), while Nativism insists that the core of linguistic knowledge, such as Merge and an inventory of formal features of Universal Grammar, are innate (Mao and Dai, 2019). Therefore, Chomskian nativists highlight more the internal mechanisms rooted in human neurobiological endowment but without excluding the roles of external factors (cf. Chomsky, 2005; Berwick and Chomsky, 2016; Mao, 2023).

^4^One reviewer points out whether “grammatical” means exactly “syntactic” under the framework of Figure 1 that is based on the complementary relationship between grammatical and pragmatic competence as Chomsky (1977) defines since “grammar” denotes a systematic ambiguity historically, viz., it refers to both the actual language internal to the person and the theory of that language constructed by the linguists [Chomsky p.c., see Note 4 in Mao (2020, p. 609)]. To clarify this ambiguity, Chomsky (1986) creates I-language (“I” stands for “internal”, “intentional”, and “individual”) for the person's actual language, keeping the term “grammar” for the linguists' theory. Because grammar is a theory that is created by linguists to describe I-language, it is supposed to explain the phenomena in syntax, semantics, phonology, morphology, and philosophical/ linguistic pragmatics of I-language. In this case, just like Hauser et al.'s (2002) decomposition of the Faculty of Language (FL) into FLN (the Faculty of Language in a narrow sense, i.e., syntactic submodule) and FLB (the Faculty of Language in a broad sense; including syntactic, semantic, phonological-phonetic submodules, but unclear whether FLB consists of pragmatic submodule), “grammar” can be understood in both narrow and broad senses. Along this vein, under the framework of IMPC, grammar or grammatical competence can be understood both narrowly and broadly, that is, either equalizing syntactic competence or referring to all kinds of sub-competence within I-language. It is because pragmatic competence is partially defined by IMPC as interactions among various submodules of I-language, such as syntactic, semantic, pragmatic, and phonological-phonetic submodules. That is, “pragmatic competence is an innate capacity that is genetically based, facilitating human's use of I-languages (based on modular interactions within I-language) for thinking (including silent communication) and utilization of linguistic representations generated by I-language, along with pragmatic knowledge, for successful sociocultural communication” (Mao, 2020, p. 627).

^5^In certain extreme situations, such as some irreversible inadequacies occurring in individuals, the abstract thinking can be disrupted, originating from the dysfunction of interactions within organism-internal submodules (i.e., A1↮A2) and/or their failed interactions with outside contexts [i.e., (A1 → A2)↮B or (A1↮A2)↮B in Figure 1].

^6^The detailed glossing of Japanese sentences in (2) is listed as follows:

a. 彼女は      また     病に           なった
she-topic       again    sick-dative    became
“She              felt       sick                again.”

b. 彼女は    また      病に           なった    のではない.
she-topic      again    sick-dative    became    nominalizer-copula
                                                                       -negative
“She             did        not feel          sick          again.”

^7^The gloss for Japanese sentence in (3) goes as follows:

a. xが                体               不良です
x-nominative    health            poor polite
“She                  is in poor      health condition.”

^8^Wiltschko (2022) also explored this type of interaction between syntax and pragmatics, and she explained it as evidence for “the distributed pragmatic meanings.”

^9^This conclusion seems to be congruent with what of Gernsbacher and Yergeau (2019), that is, even if it is not easy for autistic individuals to refer to others' mind sets, it is not safe to say that they entirely lack a theory of mind.

^10^As one reviewer points out, there is the third possibility to explain the unsuccessful interaction between Part A and Part B in Example (4), viz., the unsuccessful interaction among syntax (A1), semantics (A2), and the outside context, namely, (A1↮A2) ↮B (cf. Figure 1 and Note 5). We think that the third possibility does exist, and we actually describe it in Note 5 and further discuss the extreme possibility during our late analysis of the unsuccessful authentic communication between autistic and non-autistic people via PCEC in this paper. However, the reason we do not specifically discuss the third possibility when analyzing the silent communication between autistic and non-autistic people via IPC is that we follow Geurts et al.'s (2020) conclusion, viz., even autistic children with low verbal ability can interpret indirect requests (as the reviewer mentioned, it is possible that autistic individuals might carry out indirect requests as a result of previous experience (practice and guidance from adults)). Along this vein, we think Part B—the autistic individual in Example (4)—resorts to her/his IPC to think about the pragmatic meaning even if she/he does not directly communicate with Part A. That is, the situation can be described as (A1 → A2) ↮B.

^11^One of the reviewers reminds us of the heterogeneity or multidimensional manifestations of autism when proposing a general mental model or mechanism to probe into the unsuccessful interaction between autistic and non-autistic people. In fact, as for the heterogenous and multidimensional manifestations of ASD, we are in the same boat with the reviewer. Confronting the complex situations, we have been thinking about whether we can propose a general mental mechanism or model underlying pragmatic processing or human language use and explain why autistic people (or non-autistic people) encounter sociopragmatic and pragmalinguistic problems. In this way, the model that describes the route map of linguistic computations enables us to identify the heterogeneous manifestations of autistic individuals during their communication with non-autistic partners. The main reason we try to propose the general mental model or mechanism for language use to elucidate the sociopragmatic and pragmalinguistic problems encountered by autistic and non-autistic individuals is that language disorders might originate from the problematic operation of the general mental model or mechanism for language use, and the severity of language disorders is closely related to the severity in the operation of the mental model or mechanism, and the divergences in the operation indicate the heterogeneity of autistic language use. For example, autistic individuals with low verbal ability (Baron-Cohen, 2000) may suffer from more serious problems in the operation of the mental model or mechanism that is based on the interactions among linguistic submodules in FLB (see (A1 → A2) in Figure 1) and between all the internal linguistic submodules and outside contexts (see (A1 → A2) ↔B in Figure 1). Specifically, the problemantic operation of the mental model is exhibited by the dysfunction of interactions within organism-internal submodules (A1↮A2) and/or their failed interactions with outside contexts [(A1 → A2) ↮B or (A1↮A2) ↮B in Figure 1] (see Section 3). These situations might indicate the heterogeneity or multidimensional manifestations of autism. In this case, the advantage of our proposal is that we have the specific route map to measure or identify the “potential social barriers that individuals with autism spectrum disorder encounter in their communication with their surroundings” (comment from the reviewer).
